# Single segment of spleen autotransplantation, after splenectomy for trauma, can restore splenic functions

**DOI:** 10.1186/s13017-020-00299-z

**Published:** 2020-03-04

**Authors:** Adriana Toro, Nunziatina Laura Parrinello, Elena Schembari, Maurizio Mannino, Giuseppe Corsale, Anna Triolo, Filippo Palermo, Alessandra Romano, Francesco Di Raimondo, Isidoro Di Carlo

**Affiliations:** 1 Department of General Surgery, E. Muscatello Hospital, Augusta, SR Italy; 2 Division of Hematology, AOU Policlinico Vittorio Emanuele, University of Catania, Catania, Italy; 3 Department of Radiology, Cannizzaro Hospital, Catania, Italy; 4 Department of Clinical and Experimental Medicine, University of Catania, Catania, Italy; 5 Department of Surgical Sciences and Advanced Technologies “GF Ingrassia”, University of Catania, Cannizzaro Hospital, Via Messina 829, 95126 Catania, Italy

**Keywords:** Spleen, Abdominal trauma, Splenic function, Splenic autotransplantation, Splenectomy

## Abstract

**Background:**

Splenectomy is sometimes necessary after abdominal trauma, but splenectomized patients are at risk of sepsis due to impaired immunological functions.

To overcome this risk, autotransplantation of the spleen by using a new technique has been proposed, but so far, a demonstration of functionality of the transplanted tissue is lacking.

**Methods:**

We therefore evaluated 5 patients who underwent a splenic autotransplant in comparison with 5 splenectomized patients without splenic autotransplant and 7 normal subjects.

**Results:**

We confirmed that the patients not undergoing autotransplantation, when compared to normal subjects, had a higher platelet count, higher percentage of micronucleated reticulocytes (*p* = 0.002), increased levels of naive B lymphocytes (*p* = 0.01), a defect of class-switched memory (*p* = 0.001) and class-unswitched memory B cells (*p* = 0.002), and increased levels of PD1 on T lymphocytes CD8+ (*p* = 0.08). In contrast, no significant differences for any of the abovementioned parameters were recorded between patients who underwent spleen autotransplantation and normal subjects.

**Conclusion:**

These findings suggest that splenic autotransplantation is able to restore an adequate hemocatheretic activity as well as recover the immunological deficit after splenectomy.

## Background

The fragile structure of the spleen is frequently damaged during abdominal trauma, accounting for 25% of solid organ damage. In these patients, mortality ranges between 7% and 18% [1]. Although many techniques can be used in cases of severe splenic trauma, splenectomy is still the safest method to save the patient’s life. However, splenectomy definitively impairs the immune and hematological function of the patient after the procedure [2]. The worst complication related to the impairment of immunological functions following splenectomy is overwhelming post splenectomy infection (OPSI), a redoubtable condition of sepsis that can be lethal for the patient [3].

To avoid this complication while maintaining the immunological function of the spleen after splenectomy, spleen autotransplantation was first proposed in 1946 [4] via a technique based on multiple slices of spleen fixed into the omentum. However, many complications have been reported with this method as torsion of the omentum with consequent necrosis of the implant, chronic anemia, postoperative intestinal obstruction, and subphrenic abscesses due to necrosis of the implanted tissue. For this reasons, this autotransplantation technique has not been further developed or widely used [5]. In 1977, it was reported that non-operational management (NOM) can be performed in the event of splenic trauma. Today, whenever possible, NOM is the best choice for preserving the immunological functions of all trauma patients. But there are some cases where splenectomy cannot be avoided [6].

Recently, we reported a new technique that is able to maintain the vitality of a single fragment of transplanted splenic tissue. This transplant was located in the native position of the spleen, avoiding all the complications, of the previous techniques, related to the number and the omental position of the transplanted slices. This technique in the preliminary report was found to be completely free of complications [7].

The purpose of this manuscript is to report the clinical, radiological, hematological, and immunological status of the patients with a reasonably long follow-up period after undergoing this procedure during splenectomy.

## Materials and methods

The study is a retrospective study analyzed the findings obtained from 5 patients (group A) that underwent splenectomy with concomitant autotransplantation of the spleen, who was treated between 2010 and 2017 at the Department of Surgical Sciences and Advanced Technologies, “GF Ingrassia” of the University of Catania. This group was compared with a similar group of patients treated with splenectomy without autotransplantation (group B), while a third group of age- and matched-health volunteers was used as a normal control (group C).

### General and surgical data

Sex, age, comorbidity, cause for splenectomy, indication for splenectomy with grade according to the American Association Surgeons Trauma (ASST) [8], year of surgical procedure, duration of surgical procedure, blood loss, postoperative complications, length of hospital stay, and platelet count were analyzed for groups A and B. For group C, only sex and age were available. No subjects refused authorization to use their medical records for research, and all subjects provided their consent according to the Declaration of Helsinki.

Furthermore, patients in group A were studied from the morphological point of view with computed tomography (CT) scans during follow-up after the surgery.

### Surgical technique

The greater omentum is pedunculated in its left lateral portion, and a single segment of splenic tissue approximately 4 × 3 × 2 cm in size and of 35 g of weight is positioned in a pouch created at the lower edge of the pedunculated omentum. Using distant stitches of Prolene® 4-0, knotted without massive traction, the pouch is closed with a single segment of the spleen inside. The omental peduncle containing the splenic tissue is then anchored with 3 separate Prolene® 4-0 stitches in the parietal peritoneum, immediately below the left side of the diaphragm. This location is chosen to respect the physiological position of the spleen and to avoid torsion of the omentum.

### Radiological data

All of the patients in group A underwent a CT scan with a multidetector, obtaining images during the basal, arterial, and portal phases. Arterial acquisition was performed with the aid of the “smart-prep” with reference to the level of the celiac tripod. Layer thickness during acquisition was 1.25 mm, and the contrast flow rate was 3.5 ml/s. The nonionic contrast concentration was 350/370 mg/ml. The optimized amount of contrast was based on age and weight. The portal phase was performed at 70 s from the beginning of the infusion; multiplane re-elaborations of the arterial and portal phases were conducted. CT scan control showed the localization and size of the autotransplanted splenic tissue by contrastographic enhancement of the splenic parenchyma by the afferent and efferent vessels.

### Hematological and immunological data

All 3 groups of patients were evaluated by multiparametric flow cytometry to measure the number of micronucleated reticulocytes (MN-RET), as a biomarker of hemocatheresis, and B and T lymphocyte subsets in the peripheral blood, as a biomarker of immune dysregulation.

Peripheral blood was collected both in EDTA and in heparin tubes and processed within 2 h from the sample collection. All samples were analyzed by a NAVIOS flow cytometer (Beckman coulter), and for each analysis, 100,000 events were acquired.

### Micronucleated reticulocytes (MN-RET)

To evaluate the micronucleated reticulocytes (MN-RET) in the peripheral blood, we followed the procedure described by Dertinger SD [9].

Briefly, the cell suspension in heparin was fixed with ultracold methanol (IT. Baker) and kept at − 75 °C until the day of flow cytometric analysis.

On the day of analysis, the blood samples were removed from the − 75 °C freezer and washed with ice cold bicarbonate-buffered saline solution; then, the samples were labeled with the monoclonal antibodies anti-CD71 FITC and anti-CD42b-PE (both from Beckman Coulter) and treated with RNase (Sigma). The monoclonal anti-CD42b was incorporated into the procedure to exclude platelets, which can interfere with the analysis. After an adequate incubation period (20 min in the dark at room temperature), 1 ml ice cold propidium iodide solution (1.25 mg PI (Sigma)/ml bicarbonate-buffered saline) was added, and the results were evaluated by using a flow cytometer.

Each sample was stained and analyzed in triplicate. The MN-RETs were identified as the fraction of erythrocytes negative for CD42b and positive for CD71 and propidium iodide.

### B cell analyses

For the phenotypic characterization of the B cell subsets, we used the Dura Clone IM B Cells kit (Beckman Coulter), a reagent panel of 8 monoclonal antibodies: IgD (FITC), CD21 (PE), CD19 (ECD), CD27 (PC7), CD24 (APC), CD38 (APC-750), IgM (Pacific Blue), and CD45 (Krome Orange), according to the manufacturer’s instructions.

After gating on the CD19 positive cells, the different expression of IgM, IgD, CD38, and CD27 allowed us to distinguish the following B-subpopulations: B-naive (CD19+CD27−IgD+), B class-switched memory (CD19+CD27+CD38−IgM−IgD−), and B-class-unswitched memory (CD19+CD27+CD38−IgM+IgD+).

### T cell analyses

To identify the different T cell subsets, we used the Dura Clone IM T Cells kit (Beckman Coulter), a reagent panel of 10 monoclonal antibodies against: CD45RA (FITC), CCR7 (CD197) (PE), CD28 (ECD), PD1 (PC5.5), CD27 (PC7), CD4 (APC), CD8 (A700), CD3 (APC-750), CD57 (Pacific Blue), and CD45 (Krome Orange) according to the manufacturer’s instructions. From the gate on CD3 positive T lymphocytes, we identified the levels of CD4+ and CD8+ T lymphocytes; therefore, we evaluated the expression of PD1 on these two cell populations. Based on the different expression of CCR7 and CD45RA, we identified the following subsets of CD4+ T lymphocytes: naive T cells (CD4+CCR7+CD45RA+), central memory T cells (CD4+CCR7+CD45RA−), and effector memory T cells (CD4+CCR7−CD45RA−).

### Statistical analysis data

A statistical analysis was performed on the data collected from the 17 patients enrolled in this study. For qualitative data, the absolute frequency and the percentage were calculated. For the normally distributed quantitative data, median and standard deviation were calculated. For the not normally distributed quantitative data, median and interquartile range were evaluated.

For comparison between groups, the Fisher test was performed for qualitative data, and ANOVA was performed for quantitative, normally distributed data; ANOVA followed by a Newman-Keuls multiple comparison test. For the not normally distributed data, the Kruskal-Wallis test was used followed by the Dum test. The values are expressed as median (25th and 75th percentiles). *p* values < 0.05 were considered statistically significant.

## Results

### Clinical results

Seventeen patients were included in the present study: five patients were in both groups A and B and 7 patients were in group C.

**Fig. 1:**
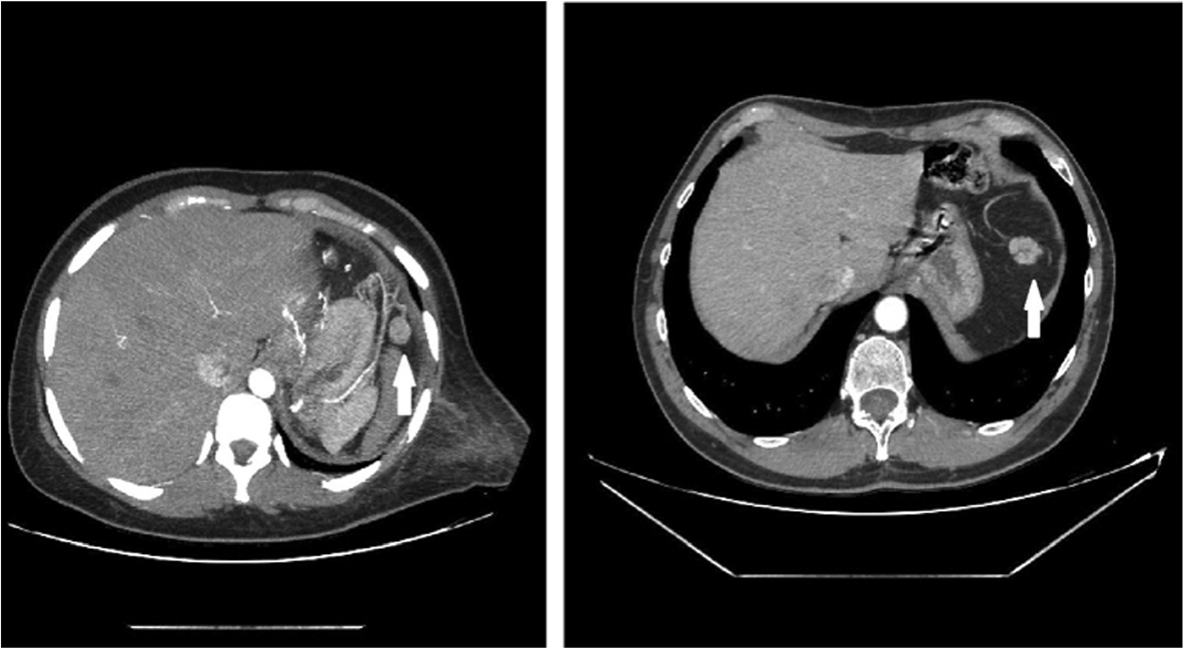
CT-scan images of two of the patients who underwent spleen autotransplantation. White arrows show the position of the new growth of the transplanted splenic parenchyma

A total of 7 patients received a spleen autotransplantation after splenectomy. However, 2 were not included in this study because 1 patient died of AIDS; the other patient is currently living in a foreign country, and it was not possible to contact him. All the characteristics of the group A are summarized in Table 1. All patients were reported to have an IV–V grade spleen trauma according to the AAST classification (Fig. 1).

**Table 1:** Characteristics of the three groups of patients

Patient	Sex	Age	Comorbidities	Cause and indication for splenectomy	Time surgical procedure	Transfusions	Blood loss	Postoperative complications	Number of platelets	Year of the splenectomy	Thrombocytosis postoperative	The size of the autotransplanted splenic tissue
1A	28	F	Bipolar syndrome	Car accident	90 min	4 bags	1000 ml	None	570,000	2017	Yes	24 × 22 mm
2A	52	F	Retinitis pigmentosa	Car accident	105 min	3 bags	500 ml	None	398,000	2010	No	15 × 10 mm
3A	54	M	None	Car accident	120 min	4 bags	2000 ml	None	318,000	2010	No	25 × 25 mm
4A	55	F	Diabetes, Hypertension	Accidental fall	90 min	2 bags	500 ml	Pulmonary thickening	245,000	2015	No	43 × 37 mm
5A	46	M	None	Accidental fall	120 min	4 bags	1000 ml	None	379,000	2012	No	15 × 22 mm
1B	26	M	None	Skiing accident	95 min	None	500 ml	None	404,000	2015	Yes	
2B	25	F	None	Car accident	140 min	None	1000 ml	None	437,000	2017	Yes	
3B	56	F	Deaf-mute, previous stroke	Car accident	155 min	4 bags	500 ml	None	581,000	2011	Yes	
4B	24	M	None	Car accident	185 min	2 bags	1000 ml	None	424,000	2017	Yes	
5B	54	F	Rheumatoid arthritis	Car accident	70 min	None	1000 ml	Pulmonary thickening	422,000	2016	Yes	
1C	41	F							229,000			
2C	52	M							206,000			
3C	30	F							299,000			
4C	28	F							216,000			
5C	31	M							200,000			
6C	30	F							219,000			
7C	29	M							231,000

All the characteristics of the group B are summarized in Table 1. All patients were reported to have an IV–V grade on the AAST classification. All the characteristics of the group C are summarized in Table 1.

No significant differences were recorded among patients in the three groups for sex and age and between groups A and B for indications for splenectomy, duration of the surgical procedure, blood loss, postoperative complications, or length of hospital stay.

### Hematological results

#### Micronucleated reticulocytes

Compared to group C (normal control), group B patients (not autotransplanted) had higher median levels of MN-RET (*p* = 0.002), while the patients in group A (autotransplanted) had levels of MN-RET that did not differ significantly from group C (Table 2).

**Table 2:** Statistical analysis of cell populations in the peripheral blood

Cell populations compared	Group A	Group B	Group C	*p* value
% MN-RET	1.3 (1–1.6)	2.7 (2.1–3.4)	0.7 (0.4–1.3)	B vs C *p* = 0.002 A vs C *p* = 0.07
B cell populations				
% B cells CD19+	10.5 (7.4–14.4)	10.3 (5.85–12.4)	8.4 (6.7–9.7)	B vs C *p* = 0.4 A vs C *p* = 0.1
% B cells naive CD19+CD27-IgD+	58 (53.5–62.6)	64.5 (58–70.9)	54.6 (46.2–59.2)	B vs C *p* = 0.01 A vs C *p* = 0.4
% B cells Class-switched memory CD19+ IgD-IgM- CD38-CD27+	15 (11.1–20.6)	12.8 (10.5–14)	19 (16.8–21.4)	B vs C *p* = 0.001 A vs C *p* = 0.06
% B cells Class-unswitched memory CD19+ IgD+IgM+CD38-CD27+	18.7 (14.5–23.3)	15.4 (11.8–18.5)	22.6 (20.1–25.8)	B vs C *p* = 0.002 A vs C *p* = 0.05
T cell populations				
% T cells CD3+	62(53.5–75.1)	75.3(56.3–79.6)	76.8(60.1–82.1)	B vs C p = 0.6 A vs C p = 0.4
% T cells CD4+	62.7(59.9–67.2)	61.8(53.4–67.2)	57.5(55–65)	B vs C p = 0.6 A vs C p = 0.4
% T cells CD8+	30(24–31.6)	30.7(24.6–35.3)	32.1(28.6–35)	B vs C p = 0.5 A vs C p = 0.1
% T cells CD4+PD1+	20.5(13.1–29.7)	29.7(26.7–30.5)	23.5(13.1–25.6)	B vs C p = 0.05 A vs C p = 0.8
% T cells CD8+PD1+	22.6(18.9–27.9)	30 (28.3–32.9)	19.3(16.3–26.1)	B vs C p = 0.008 A vs C p = 0.3
% Naive-T cells CD4+CCR7+CD45RA+	26.9(20.6–37.4)	37.1(31.6–48.2)	27.3 (24–39.4)	B vs C p = 0.2 A vs C p = 0.6
% effector memory T cells CD4+CCR7−CD45RA−	22.7 (14.5–29.3)	15.8(11.1–25.7)	22.2(14.9–24.7)	B vs C p = 0.4 A vs C p = 0.6
% central memory T cells CD4+CCR7+CD45RA-	48.6 (42.5–55.8)	42(34.1–43.2)	46.5(40.1–58.2)	B vs C p = 0.1 A vs C p = 0.7

Group A: Autotransplantation splenic tissue

Group B: Splenectomy

Group C: Healthy volunteers

Values are expressed as median (25th and 75th percentiles)

#### B cell populations

Among the three groups of patients studied, no differences were observed in the percentage of CD19 positive B lymphocytes. However, we found that group B patients had increased levels of naive B lymphocytes (*p* = 0.01) and a defect in class-switched memory (*p* = 0.001) and class-unswitched memory B cells (*p* = 0.002) compared to group C. In contrast, the distribution of the abovementioned B cell populations did not differ between groups A and C (Table 2).

#### T cell populations

The proportions of the CD3+ T lymphocytes and the CD3+CD4+ and CD3+CD8+ T-subsets were comparable in the three groups. Similarly, we did not observe significant differences in the distribution of various subpopulations of CD4+ T lymphocytes: naive T cells (CD4+CCR7+CD45RA+), central memory T cells (CD4+CCR7+CD45RA−), or effector memory T cells (CD4+CCR7−CD45RA−). However, we found that patients in group B had increased levels of PD1 on T lymphocytes CD8+ (*p* = 0.08) and T lymphocytes CD4+ (*p* = 0.05) compared to group C, while no significant differences were found between groups A and C (Table 2).

## Discussion

As WSES guidelines currently suggest NOM, including splenic artery embolization, has recently been extended to all stable patients, despite AAST grade [10], but this treatment can only be provided in a highly specialized trauma center. In peripheral hospitals worldwide, NOM for grade IV–V AAST remains a challenge, and frequently, splenectomy is considered the safer procedure both for the patient and surgeon [2].

OPSI is the most important complication after splenectomy, and it is often fatal. This complication occurs more frequently within 2 years of splenectomy [11]. In 1999, a study showed that spleen autotransplantation after splenectomy can play an important role in the immune process [12]. Furthermore, in our group of patients with autotransplant, we recorded only a patient (excluded from the present study) who died from HIV-related complications, and not for OPSI, 5 years after the autotransplant [13].

Currently, there are no studies that compared the immunological function of patients undergoing splenectomy with autologous transplantation with or without vaccination. The present study, with the limitation of the small number of patients, suggests that vaccinations may not be necessary when there has been restoration of immunological functions. The goal of spleen autotransplantation is to maintain the splenic function when splenectomy is mandatory. Many sites have been used to implant the spleen slices: the omentum, peritoneal cavity, retroperitoneum, intraportal, abdominal muscles, axillae, and liver [14]. But all adopted techniques have related complications, and for these reasons, they have been abandoned. In our patients, torsion of the implant cannot occur because the omental pouch with the autotransplanted spleen tissue is fixed and located in the native spleen position. No other complications have been recorded in our group of transplanted patients submitted to our technique, probably due to the paucity of a single portion of spleen transplanted.

Encouraged by these results, we have continued to investigate the vitality of these transplanted splenic tissues and at the same time their functionality.

The CT scan clearly shows the formation of newly formed vessels representing the arterial and venous inflow and outflow of the splenic tissue. Platelet counts returned to normal values in most patients undergoing spleen autotransplantation. In contrast, patients in the group who underwent splenectomy without autotransplantation continued to have elevated platelet counts, indicating a lack of the spleen emocatheretic activity, although the difference between the groups was not statistically significant, which was probably due to the small number of patients.

Our study has been focused on immunological data. As expected, the splenectomized patients (group B) had increased levels of MN-RET, suggesting that these patients have an altered hemocatheretic activity, confirming data reported in the literature [15]. In contrast, patients undergoing spleen autotransplantation had an MN-RET count comparable to healthy subjects (group C), indicating that in these patients, the hemocatheretic activity of the spleen has been restored.

Splenectomy has important consequences for the homeostasis of the immune system, and in particular, the B cell compartment seems to be the most involved. Our study confirmed the many observations regarding the impairment of B lymphocytes in these patients [16], showing that splenectomized patients (group B) have increased levels of B-naive and reduced levels of class-switched and unswitched memory B cells. In contrast, the autotransplanted patients did not present any significant differences for all of the evaluated B lymphocyte subsets compared to normal subjects.

Our study therefore confirms the role of the spleen in the maturation of B lymphocytes. The spleen is considered the site at which newly formed B lymphocytes are induced to mature into IgD recirculating B cells, the population that is able to initiate the humoral immune responses. In normal subjects, the B-naive cells rapidly recognize the antigen, proliferate and differentiate into short-lived plasma cells or germinal center B cells [16], thus contributing to T cell-dependent immune responses and leading to germinal center formation in follicles of secondary lymphoid organs. B cell interactions with follicular helper T cells represent a crucial step in the production of their memory B cell counterparts or plasma cells that produce high affinity, somatically mutated, class-switched antibodies [17]. An impairment of maturation may be responsible for the reduced immunological response in splenectomized patients that is restored in patients undergoing spleen autotransplantation.

We also found that spleen autotransplantation was able to mitigate the reduction of memory B cells that is observed in splenectomized patients. Memory B cells are generated in germinal centers (GC) and contribute to serological immunity by quickly differentiating into plasma cells and performing faster and more rapid responses in the clearance of the pathogen than naive B cells [18]. These cells represent a highly effective mechanism of protection against infections. Memory B cells are reduced in the peripheral blood of children and adults who are congenitally asplenic [19], and these individuals are most susceptible to infection with encapsulated bacteria [20]. Abnormal B cell homeostasis is found with certain immune deficits [21], in which a functional GC response is altered, and in some systemic autoimmune disorders [22]. Patients affected by chronic granulomatous disease (CGD) present with lower levels of memory B cells and higher levels of naive B cells [23].

By evaluating the T lymphocytes, we found that in splenectomized patients, but not in autotransplanted patients, an increase in the PD1 marker on both CD8+ and CD4+ T lymphocytes.

Programmed cell death-1 (PD-1) is an immunoreceptor belonging to the B7-CD28 superfamily that delivers negative signals when it interacts with its ligand (PD-L1) [24]. It is involved in the regulation of the mechanisms of T cell tolerance. Blocking of the PD-1/PD-L1 axis increases the response against antigens presented by dendritic cells, and it is well recognized that PD-1 is a major immune checkpoint that prevents autoimmunity [25]. However, high PD-1 expression on the surface of T cells reduces the ability of these cells to eliminate cancer and infectious diseases. It has been demonstrated that septic patients have high expression of PD-1 on T lymphocytes [26].

More important, many tumor cells express PD-L1 on their surface, and this is a clear mechanism of tumor escape from the control of immune cells. T lymphocytes in the tumor environment are often PD-1 positive and are defined as exhausted lymphocytes, indicating a poor responsive status of T cells, with decreased production of effective cytokines and a lack of cytotoxic activity [27]. In fact, targeting the PD-1 immune checkpoint has shown significant clinical efficacy in the treatment of many advanced cancers resistant to conventional chemotherapy [28].

## Conclusion

Our observations confirmed that splenectomized patients have an immunological impairment of B and T lymphocytes as well as a reduced hemocatheretic activity. These alterations were not found in patients who underwent splenic autotransplantation but who instead appear both from the immunological point of view and in regard to hemocatheretic activity more similar to healthy subjects, thus suggesting that splenic autotransplantation is able to recover the immunological deficit as well as to restore an adequate hemocatheretic activity.

However, this is a study with a limited number of patients. With these preliminary results, a large multicenter study can now be proposed.

## Data Availability

Please contact the authors for data requests

## Abbreviations

ASST: American Association Surgeons Trauma
CGD: Chronic granulomatous disease
CT: Computed tomography
MN-RET: Micronucleated reticulocytes
NOM: Non-operative management
OPSI: Overwhelming post-splenectomy infection
PD-1: Programmed cell death-1
PD-L1: Programmed cell death-ligand 1
SEA: Splenic arterial embolization
